# A comparative study of single-particle cryo-EM with liquid-nitrogen and liquid-helium cooling

**DOI:** 10.1107/S2052252519011503

**Published:** 2019-10-22

**Authors:** Olivia Pfeil-Gardiner, Deryck J. Mills, Janet Vonck, Werner Kuehlbrandt

**Affiliations:** a Department of Structural Biology, Max Planck Institute of Biophysics, Max-von-Laue-Strasse 3, 60438 Frankfurt, Germany

**Keywords:** cryo-EM, electron cryo-microscopy, radiation damage, helium, liquid-helium cooling, beam-induced motion, beam-induced movement, apoferritin

## Abstract

Radiation damage is the most fundamental limitation for achieving high resolution in electron cryo-microscopy (cryo-EM) of biological samples. The effects of radiation damage are reduced by liquid-helium cooling, although the use of liquid helium is more challenging than that of liquid nitrogen. To date, the benefits of liquid-nitrogen and liquid-helium cooling for single-particle cryo-EM have not been compared quantitatively. With recent technical and computational advances in cryo-EM image recording and processing, such a comparison now seems timely. This study aims to evaluate the relative merits of liquid-helium cooling in present-day single-particle analysis, taking advantage of direct electron detectors. Two data sets for recombinant mouse heavy-chain apoferritin cooled with liquid-nitrogen or liquid-helium to 85 or 17 K were collected, processed and compared. No improvement in terms of resolution or Coulomb potential map quality was found for liquid-helium cooling. Interestingly, beam-induced motion was found to be significantly higher with liquid-helium cooling, especially within the most valuable first few frames of an exposure, thus counteracting any potential benefit of better cryoprotection that liquid-helium cooling may offer for single-particle cryo-EM.

## Abstract 2

Radiation damage is the most fundamental limitation for achieving high resolution in cryo-EM, and is expected to be reduced at liquid-helium temperature. Surprisingly, cryo-EM reconstructions of apoferritin samples cooled with liquid helium showed no improvement in resolution over liquid-nitro­gen-cooled samples, but showed substantially more beam-induced particle motion.

## 1. Introduction   

Electron microscopy (EM) has become a key technique for determining the structures of biological macromolecules at high resolution (Vinothkumar & Henderson, 2016 ▸; Quentin & Raunser, 2018 ▸; Cheng, 2015 ▸; Kühlbrandt, 2014*a* ▸; Bai *et al.*, 2015 ▸; Scapin *et al.*, 2018 ▸), an essential step in understanding biological processes at the molecular level. The most fundamental and ultimately insurmountable limitation of the method is radiation damage to the sample. Biological samples tolerate only low electron doses, which limits the signal-to-noise ratio of the electron micrographs recorded. Cooling the sample reduces the effective damage per scattered electron significantly (Stark *et al.*, 1996 ▸; Fujiyoshi, 1998 ▸; Jeng & Chiu, 1984 ▸; Hayward & Glaeser, 1979 ▸), which is one reason why vitrified biological samples are routinely cooled, most often with liquid nitrogen (lN_2_, boiling temperature 77 K), for electron cryo-microscopy (cryo-EM). Studies of two-dimensional (2D) protein crystals have shown that liquid-helium (lHe) cooling (down to 4 K) limits radiation damage even further, since high-order diffraction spots of protein crystals fade more slowly at lower temperatures (Stark *et al.*, 1996 ▸; Fujiyoshi, 1998 ▸). Electron microscopes have been developed specially to allow lHe cooling (Fujiyoshi *et al.*, 1991 ▸) and a number of 2D crystal structures have been determined using them. In 1990, Henderson and coworkers determined the structure of bacteriorhodopsin to a resolution of 3.5 Å using data collected mostly at lHe temperature (Henderson *et al.*, 1990 ▸). The 3.4 Å resolution structure of light-harvesting complex II was then determined using only data acquired at lHe temperature (Kühlbrandt *et al.*, 1994 ▸). In successive years, high-resolution (≤4 Å) structures have been determined of various aquaporins (Murata *et al.*, 2000 ▸; Tani *et al.*, 2009 ▸; Hiroaki *et al.*, 2006 ▸; Gonen *et al.*, 2005 ▸), bacteriorhodopsin again (Kimura *et al.*, 1997 ▸), nicotinic acetylcholine receptor (Miyazawa *et al.*, 2003 ▸; Unwin, 2005 ▸) and glutathione transferase (Holm *et al.*, 2006 ▸), all using lHe cooling. For single-particle EM, helium cooling has been used only rarely (Sato *et al.*, 2001 ▸; Ludtke *et al.*, 2008 ▸; Jiang *et al.*, 2008 ▸), mostly because it resulted in an unexpected, and unexplained, loss of contrast (Danev & Nagayama, 2008 ▸; Zhou, 2008 ▸). In 2010, Bammes and coworkers reported a study with Fourier-transformed bright-field images of thin catalase crystals cooled with lHe, encountering ‘abnormal behaviour’ of Bragg peaks at 4 K (Bammes *et al.*, 2010 ▸). The intensities of some peaks were found to increase upon radiation before decreasing as expected. A similar behaviour had previously been observed for catalase crystals at room temperature (Unwin & Henderson, 1975 ▸). Electron cryo-tomography (cryo-ET) has revealed that biological samples cooled with lHe show extensive beam-induced movement and tend to bubble, suggesting that lHe cooling is disadvantageous for cryo-ET (Iancu *et al.*, 2006 ▸; Comolli & Downing, 2005 ▸). It is now thought that radiolytic fragments, which can diffuse out of the sample at liquid-nitrogen (lN_2_) temperature, are frozen into the sample at lower temperatures and accumulate within it, resulting in an expansion of the sample volume, with con­comitant beam-induced movement and reduced image contrast (Iancu *et al.*, 2006 ▸; Meents *et al.*, 2010 ▸). The effect was apparent at the comparatively high electron doses (a few 100 e^−^ Å^−2^) that are required for electron tomography. The doses applied in single-particle cryo-EM are significantly lower (typically around 50 e^−^ Å^−2^). To date, no systematic comparisons of lHe and lN_2_ cooling in single-particle cryo-EM have been published. In view of the recent advances in terms of detector speed and sensitivity (Kühlbrandt, 2014*b* ▸), specimen-support stability (Russo & Passmore, 2014*b* ▸) and correction for beam-induced motion by image processing (Zivanov *et al.*, 2019 ▸), which have revolutionized cryo-EM, it is possible that the previous difficulties can now be overcome and it is thus important to evaluate lHe cooling for single-particle cryo-EM.

We used mouse apoferritin expressed in *Escherichia coli* as a test specimen. In eukaryotic cells, ferritin catalyses the oxidation of iron(II) to iron(III), which is then stored in the oligomeric complex (Honarmand Ebrahimi *et al.*, 2015 ▸). The octahedral ferritin oligomer is composed of 24 subunits assembled into a hollow sphere in the apo state (Honarmand Ebrahimi *et al.*, 2015 ▸). Each subunit has a molecular weight of ∼21 kDa and is composed mainly of five α-helices, four of which form a bundle (Crichton & Declercq, 2010 ▸).

Owing to its high symmetry, apoferritin has excellent qualities for single-particle cryo-EM, as it allows 24-fold averaging and avoids issues of preferred particle orientation. In these respects it is superior to other commonly used test specimens of similar size, such as β-galactosidase, which only features fourfold symmetry and tends to orient preferentially on cryo-grids. The cryo-EM structure of apoferritin has recently been refined to resolutions of 1.65 Å (EMDB entry EMD-0144; Zivanov *et al.*, 2018 ▸) and 1.62 Å (EMDB entry EMD-9599), which are currently the highest resolutions reported for the technique, and apoferritin has been used as a test specimen in a number of methodological studies (Feng *et al.*, 2017 ▸; Fan *et al.*, 2017 ▸; Russo & Passmore, 2014*a* ▸,*b* ▸; Zivanov *et al.*, 2018 ▸; Marr *et al.*, 2014 ▸). It is becoming a new standard and as such is the appropriate sample for this study.

## 2. Materials and methods   

### 2.1. Sample preparation and data collection   

Samples of mouse heavy-chain apoferritin (UniProt P09528) at 5.9 mg ml^−1^ in buffer (20 m*M* HEPES pH 7.5, 300 m*M* NaCl) were diluted 1:4 in water and centrifuged to reduce aggregates in the supernatant. 3 µl droplets were deposited onto glow-discharged 300 mesh gold grids with an R2/2 gold support (Quantifoil). A Vitrobot Mark IV was used for blotting at 10°C and 100% humidity with 10 s blot time and a blot force of −2. Grids were plunge-frozen in liquid ethane. Images were recorded at 300 kV acceleration voltage in a JEOL 3200 FSC microscope equipped with an in-column energy filter operated at 20 eV and a Gatan K2 direct electron detector operated in counting mode. Eucentric height, focus and astigmatism were carefully adjusted and regularly controlled throughout data collection. A magnification of 30 000×, which corresponds to a calibrated pixel size of 1.12 Å, was used to collect 8 s dose-fractionated movies with 0.2 s frames at an electron flux of ∼9 e^–^ per pixel per second. Two data sets were collected from the same grid, selecting holes with suitable ice quality for imaging. For the first data set both cooling tanks of the microscope were filled with liquid nitrogen, such that the temperature readout on the microscope showed 78 K for the inner tank and 85 K for the specimen stage. In the second data collection the inner cooling tank was filled with liquid helium, producing a readout of 9 K for the inner tank and 17 K for the specimen stage. Images of lN_2_-cooled samples were collected on three different days and images of lHe-cooled samples on two different days, within sessions of 4–6 h each. The contamination rate in the JEOL column is estimated at 4–5 Å per hour. In between collections, the grid was stored in the lN_2_-cooled microscope side arm in order to reduce the buildup of amorphous ice. A total of 271 movies were collected with lN_2_ cooling and 233 movies with lHe cooling. Similar experiments were performed with horse apoferritin (Sigma) and with F_420_-reducing hydrogenase (Frh) from *Methanothermobacter marburgensis* (Mills *et al.*, 2013 ▸; Allegretti *et al.*, 2014 ▸).

### 2.2. Image processing and data analysis   

Movies were processed with *RELION*-3.0 beta (Zivanov *et al.*, 2018 ▸) using *MotionCor*2 v.1.0.0 (Zheng *et al.*, 2017 ▸) for initial motion correction and *Gctf* v.1.06 (Zhang, 2016 ▸) for the estimation of CTF parameters. The known magnification distortion of the electron microscope (2.57% at a minor axis angle of 35.3°) was not corrected because the current version of Bayesian polishing and CTF refinement in *RELION*-3 does not allow it. Particles were picked automatically using references from an initial set of manually picked particles. In order to discard false positives, a selection was performed via 2D classification. A total of 184 777 particles for the data set collected with lN_2_ cooling and 190 785 particles for the data set collected with lHe cooling were extracted using box sizes of 176 pixels. Three-dimensional maps were refined using a deposited apoferritin map (EMDB entry EMD-9599) filtered to 10 Å as an initial model. After this, Bayesian polishing and CTF refinement were iteratively applied in order to improve motion correction and CTF parameter estimation on a per-particle basis. The final resolutions of the reconstructions were estimated using two separate half sets according to the gold-standard 0.143 Fourier shell correlation (FSC) cutoff (Rosenthal & Henderson, 2003 ▸) and the FSC between the two reconstructions was calculated. In a separate refinement, all particles in the lN_2_ data set with less than 0.85 µm defocus were excluded. *UCSF Chimera* v.1.13.1 (Pettersen *et al.*, 2004 ▸) was used for visualization of the Coulomb potential maps for control and comparison of the map qualities. Random subsets of 400, 800, 1600, 3200, 6400, 12800, 25 600, 51 200 or 102 400 particles were individually refined for each data set and the inverse-squared resolutions obtained using these refinements were fitted to the natural logarithm of the number of particles using linear regression. Overall *B* factors were then calculated by dividing 2 by the regression slopes (Zivanov *et al.*, 2018 ▸). The standard errors of the regression slopes were calculated and propagated in order to estimate the errors in the *B* factors. To estimate the motion of particles throughout the movies, coordinates from the Bayesian polishing feature in *RELION*-3 (Zivanov *et al.*, 2019 ▸) were used to calculate Euclidean distances between particle positions in successive movie frames. These movements were added and averaged for all particles of each data set. Reconstructions containing only the information from single movie frames were produced by re-extracting particles from single frames while keeping the particle-orientation information from previous refinements. Per-frame *B* factors were calculated by the Bayesian polishing function within *RELION*-3.

All plots were produced with *Matplotlib* (Hunter, 2007 ▸) within Python. Figures showing Coulomb potential maps were produced in *USCF Chimera* v.1.13.1 (Pettersen *et al.*, 2004 ▸).

## 3. Results   

**Figure 1:**
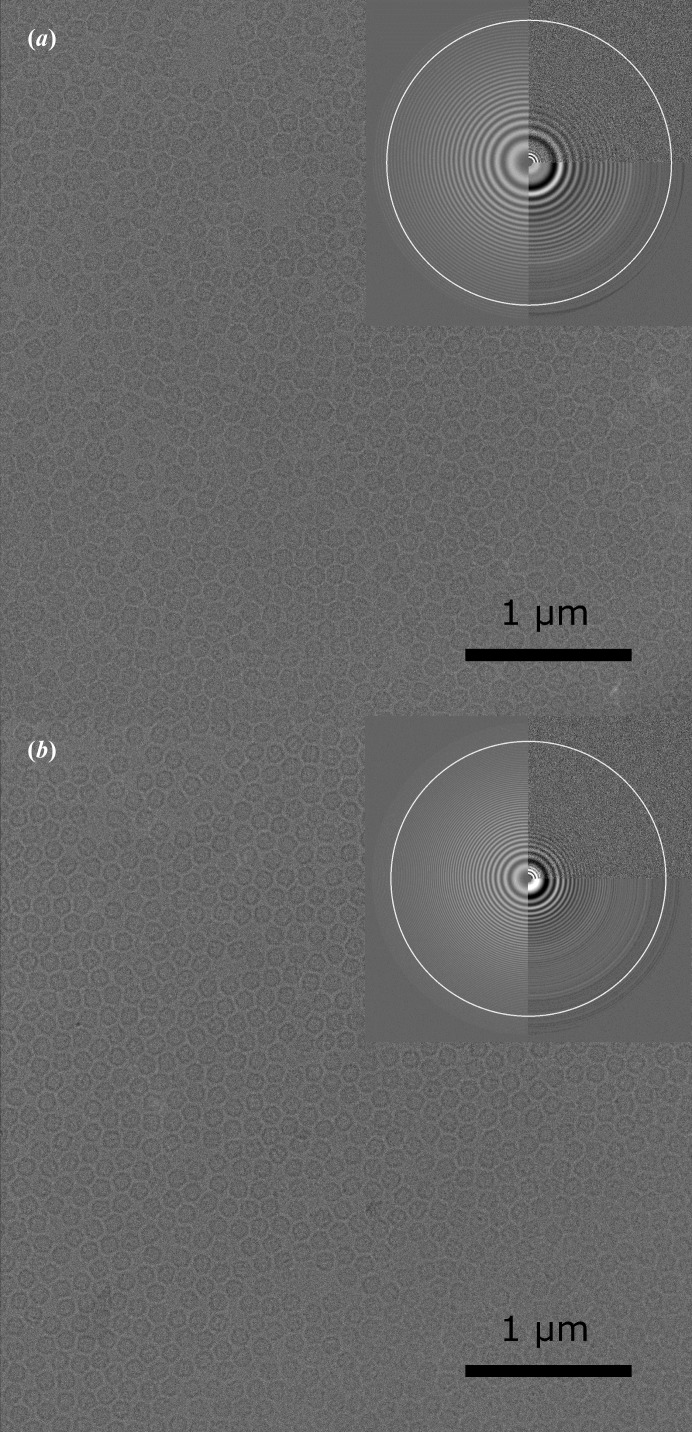
Exemplary micrographs with respective CTF estimations. (*a*) A micrograph acquired with liquid-nitrogen cooling, with an estimated defocus of 1.05 µm. The CTF goes out to 2.5 Å (white ring). (*b*) A micrograph acquired with liquid-helium cooling, with an estimated defocus of 1.99 µm. The CTF goes out to 2.6 Å (white ring).

**Figure 2:**
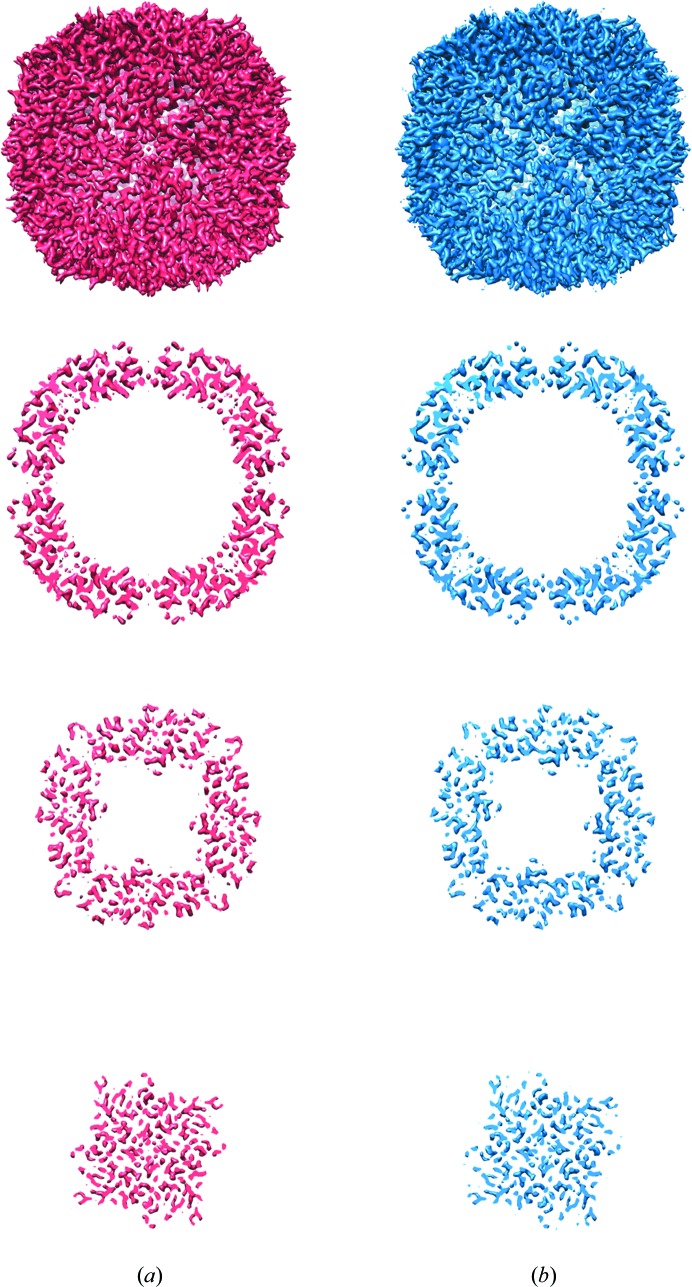
Refined cryo-EM maps and slices of mouse heavy-chain apoferritin from data acquired with liquid-nitrogen cooling (*a*) at an estimated resolution of 2.74 Å and liquid-helium cooling (*b*) at an estimated resolution of 2.78 Å.

**Figure 3:**
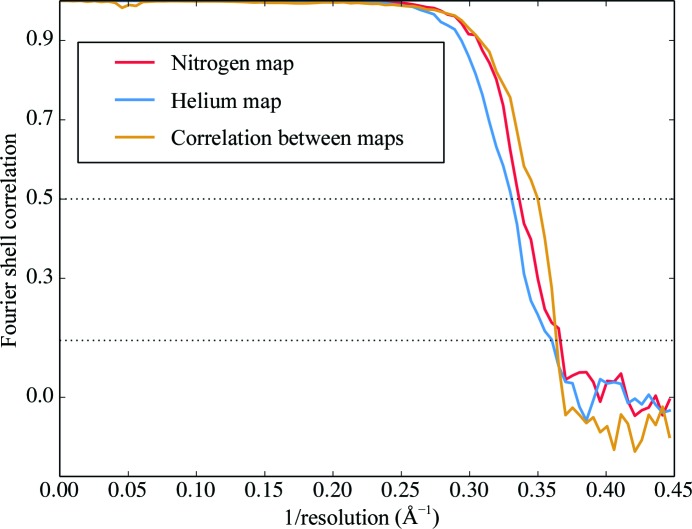
Fourier shell correlations (FSCs) of separately refined half maps from the data sets collected with lN_2_ cooling (FSC of 0.143 at 2.74 Å), lHe cooling (FSC of 0.143 at 2.78 Å) and between the two maps (FSC of 0.5 at 2.90 Å).

Two data sets of single-particle images of mouse heavy-chain apoferritin were collected at specimen temperatures of 85 K (lN_2_ cooling) and 17 K (lHe cooling) using a JEOL 3200 FSC electron microscope, in which the sample can be cooled with either lN_2_ or lHe. Exemplary micrographs and CTF estimations are shown in Fig. 1 ▸. Image processing yielded reconstructions with final resolutions of 2.74 Å for the data set collected at 85 K and 2.78 Å for the data set collected at 17 K. The reconstructed Coulomb potential maps are shown in Fig. 2 ▸ and respective Fourier shell correlations as well as the correlation between the final maps are shown in Fig. 3 ▸.

### 3.1. Comparability of data sets   

**Figure 4:**
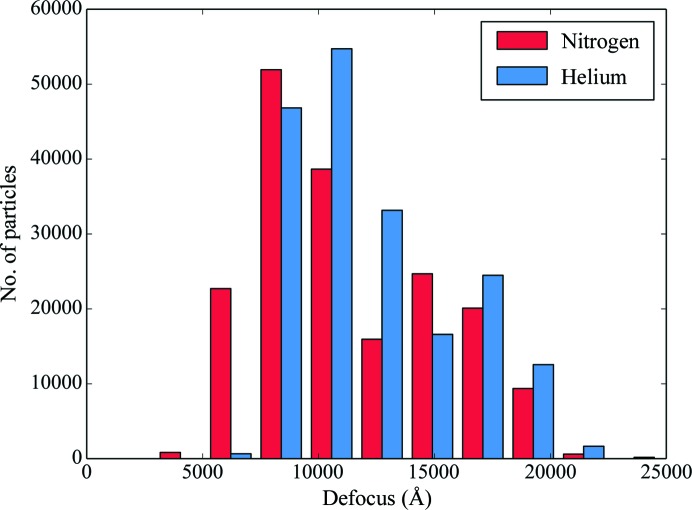
Histogram of defocus values of the particles used for reconstructions from data sets acquired with liquid-nitrogen or liquid-helium cooling.

**Figure 5:**
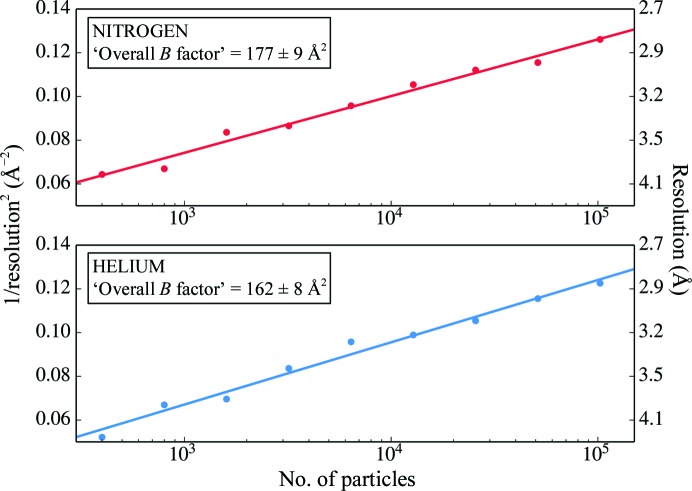
Squared inverse resolution achieved from random subsets of increasing numbers of particles (logarithmic scale) with linear fits for data sets acquired with lN_2_ and lHe cooling. From the slope, an ‘overall *B* factor’ is determined.

The resolution achieved by cryo-EM depends on a number of factors, including sample quality, ice thickness, microscope alignment and imaging conditions. It is therefore important to ascertain that the two data sets are comparable. As a first measure, all data were collected from the same grid, ensuring consistency in the sample. Fig. 4 ▸ shows the estimated defocus values for all particles used in the reconstructions. The lN_2_ data set included images recorded closer to focus, which on the one hand might contribute more high-resolution information, but on the other hand have lower contrast, either of which could bias the comparison. We ran a separate refinement that excluded all particles at less than 0.85 µm defocus from the lN_2_ data set. The resolution remained unchanged at 2.74 Å, indicating that the defocus range that we used did not affect the map quality. In Fig. 5 ▸ the squared inverse resolution of reconstructions achieved from random subsets of particles is plotted against the subset size on a logarithmic scale. Theor­etical considerations suggest this to be a linear relationship from which an ‘overall *B* factor’ can be calculated for any given data set (Rosenthal & Henderson, 2003 ▸). This *B* factor relates the achieved resolution to the number of particles used in the reconstruction. By performing linear regression, overall *B* factors of 177 ± 9 and 162 ± 8 Å^2^ were calculated for the data sets acquired with lN_2_ cooling and lHe cooling, respectively, showing that the data sets are of comparable quality. Table 1 ▸ gives a comparative overview of the two data sets.

### 3.2. Beam-induced motion and radiation damage   

**Figure 6:**
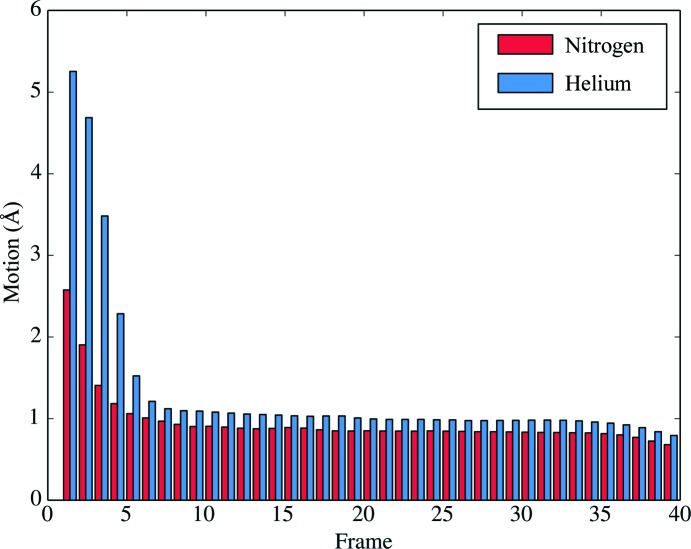
Average per-frame motion of particles imaged with lN_2_ or lHe cooling as determined by the Bayesian polishing algorithm within *RELION*-3. With lHe cooling, the motion in the first few frames is higher by a factor of more than 2.

**Figure 7:**
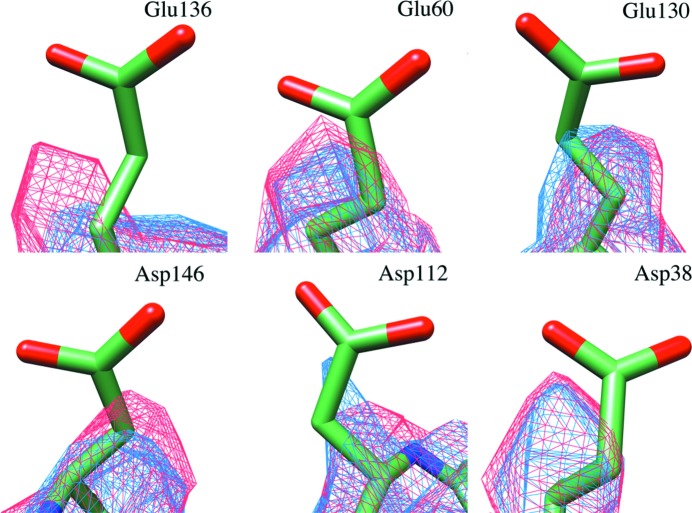
Selected carboxylate side chains showing signs of radiation damage. The reconstructed map from data acquired with lHe cooling (blue mesh) does not show better fits for the side chains of the fitted atomic model (PDB entry 3f32; Vedula *et al.*, 2009 ▸) than the map from data acquired with lN_2_ cooling (red mesh).

Fig. 6 ▸ shows that the beam-induced motion of particles in the first five or six image frames is substantially higher for lHe-cooled samples. These frames are potentially the most precious for structure determination because they have suffered least from radiation damage. The Coulomb potential maps were compared with special attention to the carboxylate side chains of glutamates and aspartates (Fig. 7 ▸), which are known to suffer first from radiation damage (Vonck & Mills, 2017 ▸). The map densities of these side chains are no better in the map derived from lHe-cooled samples, which is not surprising as this type of radiation damage already occurs at doses equivalent to less than 1 e^–^ Å^−2^ (Henderson, 1990 ▸). As expected, other less radiation-sensitive side chains did not show significant differences in density either (not shown).

**Figure 8:**
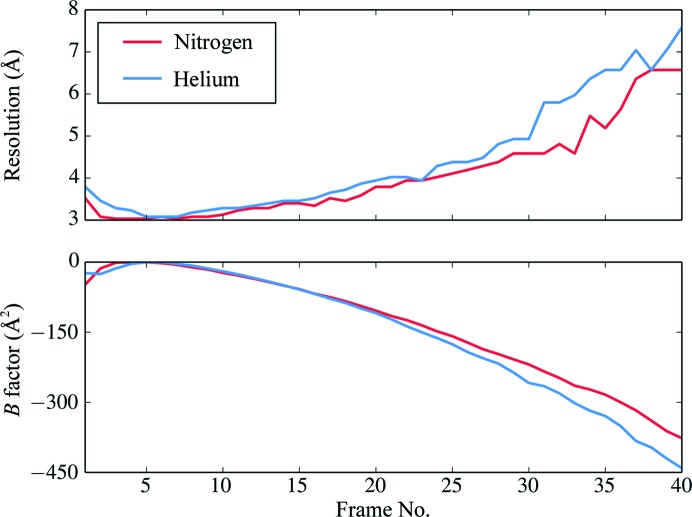
Resolutions achieved by reconstructions using only single frames of the recorded movies, keeping the optimized angles and positions from refinements using all frames (top), and per-frame *B* factors as calculated with the Bayesian polishing function within *RELION*-3 (bottom) for data sets acquired with lN_2_ or lHe cooling.

For further analysis of radiation damage, the resolutions of single-frame reconstructions and the relative per-frame *B* factors are shown in Fig. 8 ▸. These *B* factors are estimated from the Fourier ring correlation between particle images and references as a part of the damage-weighting procedure in *RELION*-3 (Zivanov *et al.*, 2019 ▸). They should not be confused with the ‘overall *B* factors’ shown in Fig. 5 ▸ or the sharpening *B* factors in Table 1 ▸. As expected, later frames had more negative, *i.e.* worse, per-frame *B* factors and lower resolution as a result of the cumulative effect of radiation damage. Surprisingly, lHe cooling proved to be worse both in terms of beam-induced movement (Fig. 6 ▸) and in terms of the resolution and decay of *B* factors beyond frame ∼20 (Fig. 8 ▸). An exception was the very first frame, which for lHe indicated a slightly better relative *B* factor for unexplained reasons.

## 4. Discussion   

The aim of this study has been to examine whether lHe cooling offers any advantage for single-particle cryo-EM. Two data sets from the same grid of recombinant mouse heavy-chain apoferritin were collected at specimen temperatures of 85 or 17 K, processed and compared.

Fig. 6 ▸ indicates two distinct phases of beam-induced movement. The first phase encompasses the first few frames for both lHe and lN_2_ cooling. For each frame the sample is exposed to a dose of 1.43 e^−^ Å^−2^ (Table 1 ▸). This low dose causes a movement of more than 5 Å in the first frame for the lHe-cooled sample. The next frames are affected progressively less, but overall beam-induced movement in this initial phase is worse by a factor of two or more for lHe compared with lN_2_ cooling. Possible causes include specimen charging (Glaeser, 2016 ▸; Russo & Henderson, 2018 ▸) and pre-existing mechanical stress frozen into the sample that is released upon radiation (Glaeser, 2016 ▸; Vinothkumar & Henderson, 2016 ▸). Both effects are likely to be stronger with lHe cooling. Charging is expected to increase owing to the lower conductivity of water and carbon at very low temperatures. Another possible cause of beam-induced movement in this early phase is the postulated collapse of vitreous water into a higher-density phase at lHe temperature that has been reported at a dose below 3 e^−^ Å^−2^ (Wright *et al.*, 2006 ▸). The rearrangement of surrounding water molecules during this density change would result in a net movement of the protein particles.

The second phase of beam-induced movement encompasses frames 7 to (in our experiments) 40. Even though in this second phase the average movement per frame (*i.e.* per 1.43 e^−^ Å^−2^) with lHe and lN_2_ is similar (Fig. 6 ▸), the per-frame *B* factor and resolution (Fig. 8 ▸) are both considerably worse for lHe beyond frame 15, at a cumulative dose of above 20 e^−^ Å^−2^. A possible explanation might be that radiolytic fragments of the protein and surrounding water, in particular molecular H_2_ (melting point 14 K, boiling point 20.3 K), are trapped in lHe-cooled samples but can diffuse into the column vacuum at lN_2_ temperature (Laufer *et al.*, 1987 ▸; Sandford & Allamandola, 1993 ▸; Flournov *et al.*, 1962 ▸). All other radiolytic products, such as oxygen, methane and ethane, are solids at lHe temperature (Vinothkumar & Henderson, 2016 ▸). Radio­lytic hydrogen may form nascent gas bubbles that expand with increasing dose, causing local movement and blurring the high-resolution signal. The nascent bubbles might correspond to an early stage of the larger bubbles observed by electron tomography of lHe-cooled samples (Iancu *et al.*, 2006 ▸).

The loss of high-resolution information is thus more severe for lHe cooling both in the initial phase of electron irradiation and at higher cumulative doses in the subsequent phase. We confirmed these findings in similar experiments with two other test specimens, Frh (Allegretti *et al.*, 2014 ▸; Mills *et al.*, 2013 ▸) and horse spleen apoferritin, which likewise indicated more beam-induced motion during the initial phase and substantially worse per-frame *B* factors in the subsequent phase for lHe cooling, as well as a better *B* factor for the very first frame (data not shown).

The best high-resolution information from dose-fraction­ated movies is expected to be found in the first few frames, where radiation damage is least severe. At present, the high-resolution signal contained in these early frames is attenuated by specimen movement, and our analysis indicates that this effect is particularly severe in lHe-cooled samples. The expected benefit of lHe cooling in terms of cryoprotection, reduced radiation damage and improved signal-to-noise ratio was not observed in this study. We conclude that at present cooling with lHe is not beneficial for single-particle cryo-EM. Once the problems associated with beam-induced specimen movement have been resolved, the potential benefit of lHe cooling for high-resolution cryo-EM should be re-examined.

## Supplementary Material

EMDB reference: apoferritin, data collected at liquid-helium temperature, EMD-4698

EMDB reference: data collected at liquid-nitrogen temperature, EMD-4701

## Figures and Tables

**Table 1:** Data collection and image processing

	Liquid-nitrogen cooling	Liquid-helium cooling
Data collection		
Microscope	JEOL 3200 FSC	JEOL 3200 FSC
Voltage (kV)	300	300
Temperature at specimen stage (K)	85	17
Camera	Gatan K2	Gatan K2
Calibrated pixel size (Å)	1.12	1.12
Electron flux (e^−^ per pixel per second)	9	9
Total exposure time (s)	8	8
No. of frames per image	40	40
Dose per frame (e^−^ Å^−2^)	1.43	1.43
Defocus range (µm)	0.30–2.11	0.66–2.47
No. of collected movies	271	233
Image processing		
Motion-correction software	*MotionCor*2	*MotionCor*2
CTF estimation software	*Gctf*	*Gctf*
Particle-selection software	*RELION*-3	*RELION*-3
No. of particle images	148777	190785
Final resolution (Å)	2.74	2.78
Applied sharpening *B* factor (Å^2^)	−126	−128
Estimated ‘overall *B* factor’ (Å^2^)	177 ± 9	162 ± 8
